# Short and Sweet: Viral 5`-UTR as a Canonical and Non-Canonical Translation Initiation Switch

**DOI:** 10.33696/immunology.3.110

**Published:** 2021

**Authors:** Brandon M. Trainor, Natalia Shcherbik

**Affiliations:** 1 Department of Cell Biology and Neuroscience, Rowan University, School of Osteopathic Medicine, 2 Medical Center Drive, Stratford, NJ 08084, USA; 2 Graduate School of Biomedical Sciences, Rowan University, 42 E. Laurel Road, Suite 2200, Stratford, NJ 08084, USA

## Abstract

The replication of viruses requires host cell functions, specifically for protein synthesis, as viruses lack their own translational machinery. Failure to translate viral mRNAs and generate viral proteins would affect the propagation and evolution of a virus. Thus, independently of their size, complexity, and genomes, viruses evolved sophisticated molecular mechanisms to hijack the translational apparatus of a host in order to recruit ribosomes for efficient protein production. One of the prevalent mechanisms of translation regulation utilized by viruses is non-canonical translation initiation. It is often governed by the 5’-untranslated regions (5’-UTRs) present upstream of a protein-coding sequence in viral mRNAs, such as internal ribosome entry sites (IRESs) and capindependent translation enhancers (CITEs). Viruses can also utilize canonical translation initiation factors of a host in non-canonical ways. Understanding strategies and mechanisms used by viruses to generate proteins is an important task, as it might help develop new therapeutic interventions. We previously have demonstrated that mRNA from the genome of the black beetle virus (BBV) of the *Nodaviridae* family contains short and unstructured 5’-UTR, which governs translation initiation as a CITE and as a canonical translational enhancer. In this Commentary, we summarize cap-dependent and cap-independent translation initiation mechanisms and further elaborate on the unique ability of the BBV mRNA 5’-UTR to switch between these two modes of translation initiation in the context of the viral life cycle. Medical implications in treating the severe acute respiratory syndrome coronavirus 2 (SARS-CoV2) infection by targeting viral 5’-UTRs are also discussed.

## Introduction

The conventional view of gene expression regulation is based on transcription control. However, a growing number of recent studies has revealed the important additional impact of translational regulation. Eukaryotic translational machinery appears to be capable of reprogramming mRNA translation to generate proteins required to maintain a healthy cellular proteostasis under physiological conditions or adapt to stress. Translation across all life species occurs in four stages: initiation, elongation, termination, and recycling. The first stage, initiation, is more highly regulated than subsequent steps regardless of cell type, thus serving as the rate-limiting step of protein synthesis. Furthermore, initiation can occur via several mechanisms, of which canonical cap-dependent translation initiation is predominant. A mounting body of evidence indicates that initiation of protein synthesis can utilize cap-independent mechanisms, which are often used by viruses. Therefore, understanding non-canonical mechanisms of translation initiation will not only advance fundamental knowledge of viral life cycle progression and host cell physiology, but is also critical for winning the battle against viral infections. In this Commentary, we discuss the role of the 5’-untranslated region (5’-UTR) from the genome of the black beetle virus as a potential regulatory switch between canonical and non-canonical modes of translation initiation.

## Canonical Cap-Dependent Translation Initiation

**Figure 1:**
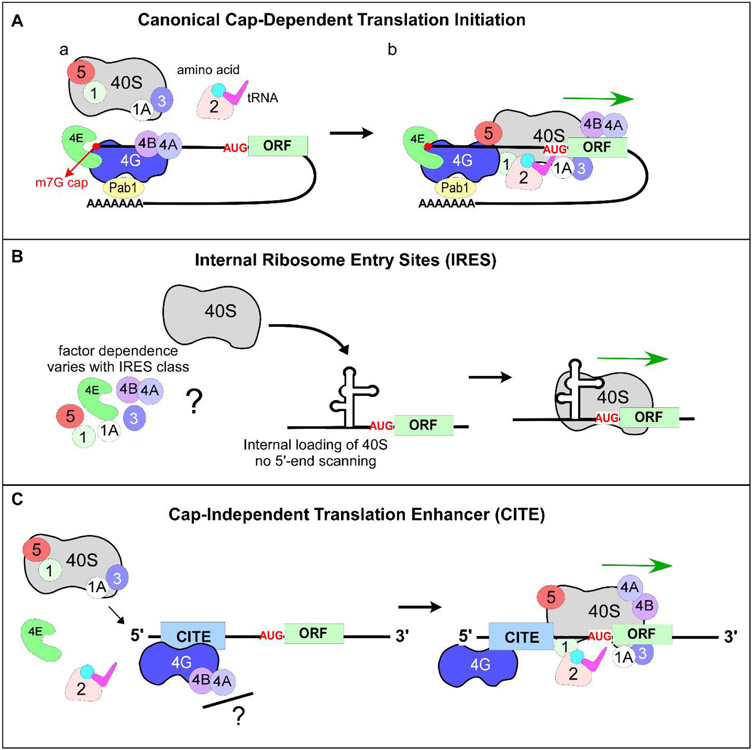
Canonical and non-canonical mechanisms of translation initiation. **(A)** Schematic representation of canonical or cap-dependent translation initiation. Translation initiation factors eIF1, eIF1A, eIF2, eIF3, eIF5, eIF4E, eIF4A, eIF4B, eIF4G are labeled as numbers. Small ribosomal subunit is marked as 40S. The 43S PIC (40S, eIF1, eIF1A, eIF3, eIF5, and eIF2•GTP•Met-tRNA_i_^Met^) is recruited to the 5’-end of mRNA via the cap-binding protein eIF4E bound to m7G-cap (red circle), and RNA helicase eIF4A is recruited to the scaffold protein eIF4G. **(B)** IRES-mediated translation initiation. Ribosomes are recruited internally (downstream of 5’-end) to mRNA and positioned adjacent to or on the initiation codon. IRES is represented as a complex secondary RNA structure. **(C)** CITE-directed translation. Translation machinery is recruited by eIF4G to the extreme 5’-end of the mRNA independently of capping. CITEs utilize 5’-end scanning similar to canonical translation but unlike IRES-mediated translation initiation mechanism. **(B and C)** Cap-binding factor eIF4E does not participate in IRES- and CITE-mediated translation initiation mechanisms.

Canonical translation initiation is a complex, multistep process that requires coordinated activity of multiple translation initiation factors (eIFs). It begins when the methyl-7-guanosine (m7G) structure is co-translationally added at the 5’-end of the mRNA transcript (Figure 1A-a). The cap serves as a recognition signal for cap-binding protein eIF4E, a member of the eIF4F family of initiation factors (which also includes eIF4G, eIF4A, and eIF4E), and stabilizes mRNA by preventing 5’-end degradation by exonucleases [1-3]. eIF4E is subsequently joined by the scaffold protein eIF4G, which recruits the RNA helicase eIF4A required for melting the secondary structure of the 5’-UTR sequence [4]. The 5’-UTRs (also known as leader sequences) are the non-coding sequences, present on the transcript upstream of the open reading frame (ORF). Cap-mediated binding of mRNA to the eIF4F complex is an essential step during canonical translation initiation, followed by interaction with the 43S pre-initiation complex (43S PIC). The 43S PIC is composed of a 40S ribosomal subunit bound to translation initiation factors eIF1, eIF1A, eIF3, eIF5, and eIF2•GTP•Met-tRNA_i_^Met^, wherein Met-tRNA_i_^Met^ is aminoacylated initiator tRNA_i_^Met^ (Figure 1A-a). Recruitment of the 43S PIC to the capped, eIF4F-bound mRNA results in the assembly of mRNA scanning-competent 48S initiation complex (48S IC) [5]. The 48S IC scans mRNA in the 5’→3’ direction until a start codon (typically AUG) is recognized (Figure 1A-b), after which eIFs (except eIF1A) dissociate and the large ribosomal subunit 60S bound to eIF5B•GTP is recruited. Hydrolysis of eIF5B•GTP results in dissociation of eIF5B•GDP and eIF1A, leading to the formation of elongation-competent ribosome 80S [6] that decodes mRNA and forms peptide bonds between amino acids, resulting in polypeptide synthesis. An additional factor, the poly-A binding protein (PABP), associates with the poly-adenine tail present on the 3’-end of most mRNAs and also interacts with capbinding proteins, thus providing communication between the 5’-end and the 3’-end of a transcript [7] (Figure 1A). These unique features allow the cell to distinguish between mRNA and other RNAs, such as degradation products or viral RNAs that may lack the 5’-m7G cap or polyA tail.

## Cap-Independent 5’-UTR-Governed Translation Initiation: IRES and CITE Elements

Although most cellular mRNAs contain the same 5’-m7G cap, translation initiation rates vary drastically [8]. This can be partly explained by 5’-UTRs, as these sequences have been shown to impact translation initiation control. 5’-UTRs vary significantly in length, sequence context, and secondary structure [9-12]. A pool of mRNAs transcribed from the same gene may differ in both length and terminal 5’-end nucleotide sequences based on the variability of the transcription start site for RNA polymerase [10,13]. Indeed, work from Gilbert’s lab demonstrated that rates of ribosome association with mRNAs differ over 1000-fold depending on the 5’-UTR sequence, further highlighting the critical role of 5’-UTRs in translation initiation control [14].

Synthesis of new proteins consumes the vast majority of a cell’s energy pool [15]. Therefore, translation must be tightly regulated in response to all conditions a cell might experience to allow adaptation to a broad range of intra- and extracellular cues. Thus, most stresses lead to an immediate reduction in protein synthesis rates, typically accompanied by global shifts in specific gene expression towards the upregulation of stress-responsive genes [16,17]. Eukaryotes downregulate translation using two mechanisms: phosphorylation of the α-subunit of eIF2 and activation of eIF4E-binding proteins (4E-bps), which inhibit the association of eIF4E with the 5’-cap [18-21]. In both cases, global protein expression is reduced; however, to survive, cells employ non-canonical mechanisms of translation initiation, which bypass the requirement of the 5’-cap structure [22]. In such cases, the 5’-UTR may be the sole determinant of translation initiation efficiency.

The most characterized type of non-canonical cap-independent translation initiation occurs via internal ribosome entry sites (IRES). First characterized within viral RNAs, IRES elements have been found within numerous eukaryotic mRNAs, predominantly in those involved in stress response [23-25]. IRES recruit a ribosome *internally* in close proximity to the AUG, as opposed to the 5’-end-dependent binding followed by 5’→3’ directional scanning of mRNA by 40S observed during canonical translation initiation [26] (Figure 1B). Some IRES elements demonstrate a remarkable ability to initiate translation with minimal requirements for canonical translation initiation factors. Furthermore, IRES range considerably in their respective size, structure, and sequence context [27]. Some IRESs have been shown to use a complex secondary structure(s) within mRNA 5’-UTR that stimulates ribosome recruitment, while other IRES elements do not rely on a secondary structure [28-31]. Interestingly, upregulation of IRES-containing transcripts has been observed in multiple organisms under conditions when canonical translation is repressed [23,32]. These observations partially explain the strategy eukaryotic cells have developed to continue synthesizing new proteins under conditions when canonical cap-dependent translation initiation is inhibited.

Another mechanism of cap-independent translation initiation uses cap-independent translation enhancers (CITEs). Similar to IRESs, CITEs bypass the requirement for the 5’-m7G cap and vary in their size, structure, location (5’-UTR vs. 3’-UTR), and translation initiation factor requirements [33-35]. However, unlike IRESs, CITEs require the 5’-end of mRNA for recruiting translational machinery, followed by mRNA scanning rather than loading a ribosome via internal entry [35]. CITEs have been identified in cellular mRNAs [36,37] and promote direct recruitment of initiation factors to the mRNA in a manner distinct from the canonical translation initiation mechanism (Figure 1C). Therefore, CITEs provide an additional cap-independent mechanism of stimulating translation initiation.

## Strategies used by Viruses to Hijack the Translational Machinery

Viruses and their hosts have undergone a co-evolutionary battle over the hosts’ translational machinery and essential translational factors [24,38]. Most viruses lack a 5’-m7G cap and/or polyA tail and are able to hijack the translational machinery of a host by employing non-canonical translation initiation mechanisms for the production of viral proteins [39,40]. Viruses owe this ability to IRES and CITE elements present in their RNAs. For example, the IRES of the intergenic region of cricket paralysis virus (CrPV, family *Discistroviridae*) promotes recruitment of the 40S subunit in a factor-independent manner [41]. Specific domains of hepatitis C virus (HCV, family *Flaviviridae*) IRES interact with the small ribosomal subunit and only two initiation factors (eIF3 and eIF5B), allowing expression of viral RNA [42]. Viruses can also utilize canonical translation initiation factors in non-canonical ways. For example, while acting as a scaffolding protein downstream of eIF4E cap binding during cap-dependent initiation, eIF4G bypasses the requirement of eIF4E during non-canonical initiation. Some viral RNAs utilize adenine-rich regions to recruit PABP to the 5’-UTR directly or to the A-rich (but not poly-adenylated) 3’-UTR, thus stimulating eIF4G recruitment and initiation complex assembly [43,44]. Although less studied than IRES elements, CITE-mediated translation initiation mechanisms can also be used by viruses [33,35,45,46].

## Dual Role of The 5’-UTR of RNA1 from Black Beetle Virus during Translation Initiation

In our work published in *The Scientific Reports* [46], we investigated the mechanism by which the 5’-UTR derived from the black beetle virus (BBV, *Nodaviridae* family) genome participates in translation initiation. BBV is a bipartite, positive-sense, single-stranded RNA virus [47] that causes paralysis in wax moth larvae but does not replicate in mammalian cells like Flock House virus (FHV) from the same family does [48]. The BBV genome is composed of two RNAs, RNA1 and RNA2, encapsidated in a single virion. RNA1 (3015 nucleotides) encodes the viral replicase (RNA-dependent RNA polymerase) required to synthesize additional genomic RNA [49] and is therefore critical for viral propagation. RNA2 (1399 nucleotides) encodes the precursor protein necessary for capsid formation. RNA1 also contains a 5’-UTR of 39 nucleotides that can stimulate translation of Coxsackievirus RNA, revealing a general translation initiation function independent of the coding portion of mRNA [50]. This short 5’-UTR was of particular interest due to its previously undescribed mechanism as a translation enhancer [51], which directs efficient translation in a wide variety of eukaryotic cells and cell extracts (i.e., insect, yeast, mammalian), suggesting that this 5’-UTR exploits a mechanism that is widely used in different eukaryotes.

To unravel how the 5’-UTR of BBV RNA1 operates during translation initiation, we devised a *Saccharomyces cerevisiae*-based cell-free translational system that allowed us to demonstrate that this short viral leader constitutes a CITE element [46]. Using mRNA reporter *Renilla* luciferase with altered sequences of the BBV RNA1 5’-UTR, we found that this regulatory element is unstructured, is required in its entirety for efficient cap-independent translation initiation, and relies on 5’-end scanning rather than on an internal entry mechanism. Using molecular and genetic approaches, we constructed yeast strains that overexpressed wild-type eIF4G1 or eIF4G1 mutants with deleted mRNA-binding domains, and used these strains to prepare translationally active lysates. Elevated eIF4G1 levels resulted in increased translational efficiency of the reporter placed under the control of BBV RNA1 5’-UTR, suggesting that this initiation factor is limiting. These results corroborated previously published studies in which yeast eIF4G1 was found to be a limiting factor in cap-independent translation initiation accomplished via IRES elements present at 5’-UTRs of yeast transcripts that encode for proteins required for pseudohyphal growth [52]. It was proposed that A-rich 5’-UTR/IRES directly interact with Pab1/PABP, which recruit eIF4G1, bypassing dependence on the cap and the cap-binding eIF4E factor. We found that for BBV RNA1 5’-UTR, eIF4G1 recruitment depends on the arginine-rich RNA-binding domains identified in [53], suggesting that this 5’-UTR exploits the RNA-binding properties of eIF4G1 to promote initiation. Whether the interaction between BBV RNA1 5’-UTR and eIF4G1 is direct remains to be determined.

What could be the biological significance of cap-independent translation initiation mediated by the BBV RNA1 5’-UTR? A previous study has shown that translation of BBV RNA1 is switched off in favor of RNA2 translation in the later stages of viral infection [54]; this is believed to promote capsid protein expression, funneling host resources towards viral encapsulation and assembly. By testing the concentration dependence of BBV RNA1 5’-UTR-mRNA on the efficiency of protein synthesis in our cell-free translation system, we detected an increase of reporter protein synthesis proportional to mRNA levels (BMT and NS, unpublished data). This suggests that BBV RNA1 might be competing with RNA2 for canonical translational machinery in late stages of viral infection. The existence of a CITE on BBV-RNA1 may therefore facilitate continual expression of replicase using an alternative, cap-independent mechanism. This might be essential for BBV propagation in its native host and allow viral replication even when translational efforts are channeled towards BBV-RNA2, providing an evolutionary advantage over viruses that lack a *cis*-acting sequence element to drive translation independently of the 5’-cap status.

**Figure 2:**
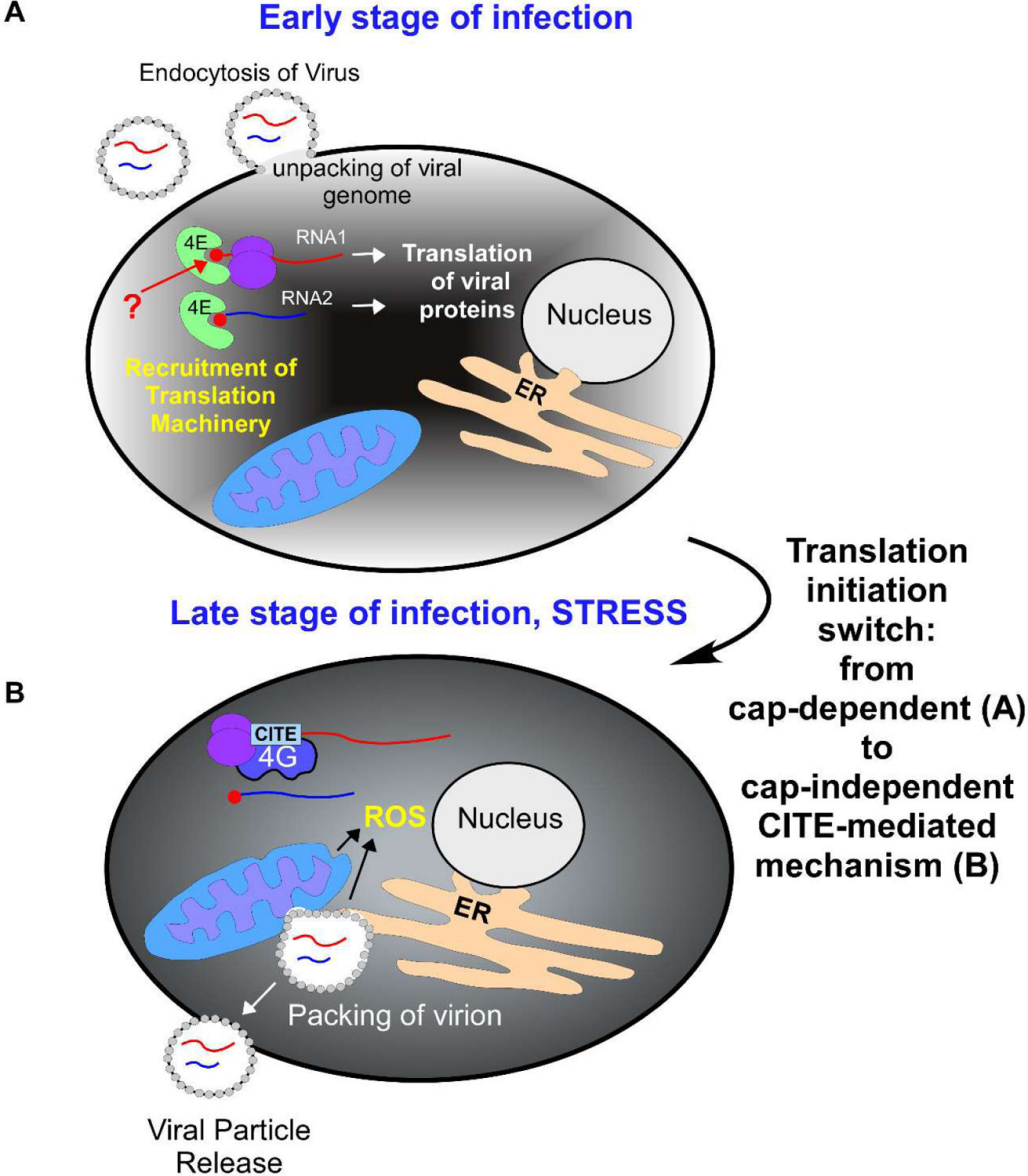
Translation initiation switch model for black beetle virus RNA1 5′-UTR CITE. During early stages of infection **(A)**, both black beetle virus (BBV) RNAs (RNA1 is shown in red, RNA2 is shown in blue) are translated via canonical cap-dependent translation initiation mechanism, in which the RNA1 5′-UTR CITE element functions as a Kozak sequence to enhance start codon selection. In later stages of BBV infection **(B)**, cap-dependent translation is downregulated due to stress induced, in part, by disruption of mitochondrial and ER membranes for virion packaging and accompanying release of reactive oxygen species (ROS). Under these conditions, BBV RNA1 5′-UTR CITE activity is switched to operate in cap-independent translation initiation.

Finally, we found that BBV RNA1 5’-UTR improves translation efficiency during canonical cap-dependent translation initiation, likely by facilitating selection of the correct start codon and thus resembling a Kozak sequence [46]. These data demonstrated the unique ability of this 5’-UTR to stimulate translation as both the CITE element and as a canonical translation enhancer [46]. Although, the capping status of BBV RNA1 remains unaddressed (unlike for BBV RNA2 [55]), the viral replicase contains a putative capping domain. Thus, it is feasible that BBV RNA1 is also capped, as it is in the closely related FHV [56]. Therefore, it is reasonable to propose that BBV RNA1 5’-UTR might function as a regulatory switch between the two modes of translation initiation (Figure 2), providing a strong adaptive advantage during infection when cap-dependent translation is downregulated due to virus-induced stress.

The ability of a viral RNA to stimulate both cap-dependent and cap-independent translation initiation would allow the virus to utilize canonical initiation immediately upon infection and later switch to non-canonical initiation upon infection-induced cellular stress (Figure 2). Indeed, infected *Drosophila* cells showed high amounts of BBV RNA1 5’-UTR-dependent protein production, eventually encompassing 40% of total cellular protein and likely causing metabolic stress [57,58]. Additionally, viruses of the *Nodaviridae* family typically infect host cells and replicate using invaginations on the outer membrane of mitochondria and endoplasmic reticulum (ER), effectively stealing part of the mitochondrial membrane for forming the viral capsid. Under such circumstances, mitochondrial perturbations could lead to increases in reactive oxygen species (ROS) or activation of the host’s antiviral response, leading to translation repression (Figure 2B) [59]. This presents an additional circumstance in which a virus would benefit from switching to a cap-independent translation mechanism to continue translating the downstream reading frame encoding its replicase.

## Viral 5’-UTRs as Antiviral Therapeutic Targets

Coronaviruses, like the severe acute respiratory syndrome coronavirus 2 (SARS-CoV2) that is currently causing an unprecedent pandemic, utilize stress-responsive pathways in host cells to assist in their replicative cycles. Although coronaviral RNAs are capped and poly-A-tailed, they may still utilize a switch-like mechanism described for BBV when canonical translation initiation is downregulated [60,61]. Understanding non-canonical mechanisms of translation initiation is therefore of the utmost importance to advance our knowledge of both normal cellular physiology and of viral infection and propagation mechanisms. Recently, strategies have been proposed to target the 5’-UTR of SARS-CoV2 RNA to inhibit the synthesis of viral proteins, thus halting replication at the earliest stages [62]. Additionally, UTRs of SARS-CoV2 genome have been recently suggested to evolve more rapidly to avoid host detection by micro RNAs, as these regions do not encode any functional elements [63].

Viral 5’-UTRs are required not only for translation initiation but also for replication. Viral polymerases recognize key features within 5’-UTRs to begin transcription, presenting another key function of these regions [64,65]. Thus, UTRs within viral RNAs may be potential future antiviral therapeutic targets that not only serve to block translation but may also curb continuous replication of the viral genome by additionally inhibiting RNA transcription [66,67].
